# Safety evaluation of a vaccine: Effect in maternal reproductive outcome and fetal anomaly frequency in rats using a leishmanial vaccine as a model

**DOI:** 10.1371/journal.pone.0172525

**Published:** 2017-03-01

**Authors:** Rafaianne Q. Moraes-Souza, Ana Paula Reinaque, Thaigra S. Soares, Ana Luiza T. Silva, Rodolfo C. Giunchetti, Maria A. S. Takano, Milena A. Akamatsu, Flávia S. Kubrusly, Fernanda Lúcio-Macarini, Isaias Raw, Dmitri Iourtov, Paulo Lee Ho, Lilian L. Bueno, Ricardo T. Fujiwara, Gustavo T. Volpato

**Affiliations:** 1 Laboratory of System Physiology and Reproductive Toxicology, Institute of Biological and Health Sciences, Federal University of Mato Grosso (UFMT) - Barra do Garças, Mato Grosso State, Brazil; 2 Laboratory of Immunology and Genomics of Parasites, Department of Parasitology, Biological Sciences Institute, Federal University of Minas Gerais (UFMG) Belo Horizonte, Minas Gerais State, Brazil; 3 Laboratory of Cell-Cell Interactions, Morphology Department, Institute of Biological Science, Federal University of Minas Gerais, Belo Horizonte, Minas Gerais, Brazil; 4 Division of Technological Development and Production (DDTP), Butantan Institute, São Paulo, Brazil; Academic Medical Centre, NETHERLANDS

## Abstract

While the immunogenic potential of the vaccination against infectious diseases was extensively shown, data on the safety assessment of recombinant proteins in vaccine formulations administered during pregnancy are still scarce. In the current study, the antigenicity of a vaccine against leishmaniasis (based on *Leishmania braziliensis* recombinant protein peroxidoxin) during pregnancy and possible maternal reproductive outcomes and fetal anomalies after immunization with a leishmanial vaccine or adjuvant alone (*Bordetella pertussis* derived MPLA adjuvant) were assessed. Rats were mated and allocated in three groups: **Control**—rats received saline; **Adjuvant**—rats received the adjuvant MPLA, and **Vaccine**—rats received the combination of MPLA and peroxidoxin. The administration was subcutaneously at the dorsal region, three times (days 0, 7, 14 of pregnancy). On day 21 of pregnancy, all rats were bled for biochemical and immunological measurements. The gravid uterus was weighed with its contents, and the fetuses were analyzed. The immunization with peroxidoxin induced a significant production of circulating IgG levels compared to other groups but caused a significant in post-implantation loss (14.7%) when compared to Control (5.0%) and Adjuvant (4.4%) groups. Furthermore, a significantly high rate of fetal visceral anomalies, such as hydronephrosis and convoluted ureter, was also observed in animals that received vaccine when compared to Control or Adjuvant groups. These data indicate the importance of safety evaluation of vaccines during pregnancy and the limited use of peroxidoxin administration during pregnancy. More importantly, the safety monitoring of immunization with MPLA derived from *Bordetella pertussis* demonstrated no reproductive outcomes associated with adjuvant administration, suggesting its safe use during pregnancy.

## Introduction

Leishmaniasis are a complex of diseases caused by protozoan parasites from more than 20 *Leishmania* species transmitted to humans by the bites of infected female phlebotomine sandflies, blood transfusion or placenta transfer of amastigotes during pregnancy [1–3]. Despite the high prevalence of the disease—estimated at 1.3 million new cases and 20,000 to 30,000 deaths every year [4]–there is no effective vaccine for control of leishmaniasis [5–7].

While vaccines against infectious diseases would be highly desirable to prevent the infection or even adverse outcome of the disease it is not clear whether such vaccines might be applicable to pregnant women during vaccination campaigns due to lack of safety data and potential risks to the fetus. Nonetheless, the maternal immunization would be of interest to prevent diseases with increased morbidity in pregnancy, their fetus or infants, offering antibodies to the children in a period when they would not respond adequately to the vaccine stimuli [8]. Of note, the clinical manifestations of leishmaniasis during pregnancy are critical as increased rates of maternal and fetal morbidity and mortality are often reported, besides most of the available drugs are not safe for use during pregnancy [9,10]. Some drugs were confirmed as safe during gestation in visceral leishmaniasis treatment, such as liposomal amphotericin B with sodium stibogluconate [11], but others treatment still require studies to prove their safety.

There are few randomized controlled studies to assess the safety of vaccines for use during pregnancy [12–14]. More importantly, the reports on safety monitoring during pregnancy to Monophosphoryl Lipid A (MPLA) adjuvant are still scarce despite the large use in several vaccine combinations [15–17]. In the current study, the antigenicity of vaccines against leishmaniasis during pregnancy and possible maternal reproductive outcomes and fetal anomalies after immunization with a leishmanial vaccine with the Peroxidoxin 1 (Pxn-1) [18,19] as a model or adjuvant alone (*Bordetella pertussis* derived MPLA adjuvant) were assessed. Our data showed that vaccination against leishmaniasis using a recombinant Pxn-1 elicited the production of specific IgG antibodies, but the vaccine induced an increased post-implantation loss and fetal anomalies, indicating the importance of safety evaluation of vaccines and the limited use of peroxidoxin administration during pregnancy. On the other hand, the safety monitoring of immunization with MPLA derived from *Bordetella pertussis* demonstrated no reproductive outcomes associated with adjuvant administration, suggesting its safe use during pregnancy.

## Materials and methods

### Cloning, expression and purification of peroxidoxin 1

Cloning, expression and purification of peroxidoxin 1 were performed as previously published [20]. Briefly, the primers used to amplify the peroxidoxin 1 gene from the *Leishmania braziliensis* genomic DNA were Pxn-1-Forward, 5′***GCTAGC****ATGCTCCGTCGTCTTGCT*, and Pxn-1-Reverse, 5′***AAGCTT****TCACATATTCTTCTCAAAAAATTCGC*. Sites for restriction enzymes (*Nhe*I and *Hind*III, respectively)–added to facilitate cloning—are shown in bold. The amplified DNA fragments were excised from the gel, purified and linked into *p*GEM-T Vector Systems (Promega, USA). Recombinant plasmid pGEM-Pxn-1 was used to transform *Escherichia coli* XL1-Blue (Phoneutria, Brazil) competent cells. Positive transformants were tested by restriction analysis with *Nhe*I and *Hind*III, and those presenting the peroxidoxin gene were propagated and used for constructing the expression vector. DNA fragments obtained from digestion of pGEM-peroxidoxin with *Nhe*I and *Hind*III were ligated into pET28a-TEV. Electrocompetent *E*. *coli* BL21 Arctic Express (DE3) (Agilent Technologies, USA) cells were transformed by electroporation using a MicroPulser Electroporation Apparatus (Bio-Rad Laboratories, USA) with the recombinant plasmid pET28a-TEV-Pxn-1. Gene insertion was confirmed by colony PCR and sequencing using T7 primers (Macrogen, South Korea).

The expression was induced in transformed *E*. *coli* by the addition of IPTG to a final concentration of 1.0 mM, and the culture was incubated for 24 h, at 12°C and 200 rpm.min^−1^. The cells were ruptured by sonication, the debris was removed by centrifugation, and the recombinant protein was purified onto a HisTrap HP affinity column connected to an ÄKTAprime chromatography system (GE Healthcare, USA). The eluted fractions containing the *r*Peroxidoxin (227 amino acids, 25.3 kDa) were concentrated in Amicon ultra 15 Centrifugal Filters 10,000 NMWL (Millipore, Germany) and further purified on a Superdex 200 gel-filtration column (GE Healthcare Life Sciences, USA).

### Vaccine preparation

Each dose of Pxn-1 vaccine comprised of 10 μg of recombinant Pxn-1 formulated with 10 μg of emulsified Monophosphoryl Lipid A (MPLA). MPLA was produced using LPS from previously detoxified whole cell pertussis vaccine, followed by organic extraction and hydrolysis as described [21].

### Experimental animals

Female Wistar rats (230–250 g) were obtained from the UFMT animal facility and were maintained under standard laboratory conditions (22±3°C, 12-h light/dark cycle) with pelleted food (Purina rat chow, Purina^®^, São Paulo, SP, Brazil) and water *ad libitum*. The procedures and animal handling were performed in accordance with the guidelines provided by the Brazilian College of Animal Experimentation in agreement with the International Guiding Principles for Biomedical Research Involving Animals promulgated by the Society for the Study of Reproduction and were approved by the Ethical Committee for Animal Research of the UFMT, Brazil (Protocol# 23108.007931/14-0).

### Mating procedure

All female rats were mated overnight. The day when sperm was found in the vaginal smear was designated as gestational day 0. The mating procedure consisted of 15 consecutive days, a period comprising approximately three estrous cycles, until a replicate number of groups were obtained. After this period, nonmated female rats were considered infertile and were discarded from the study [22].

### Experimental groups

Pregnant rats were randomly divided into three groups (n minimum = 11 animals/group): Control (immunized with 200 μl PBS), Adjuvant (200 μl PBS + 10 μg of emulsified MPLA), and Vaccine (200 μl PBS + 10 μg of emulsified MPLA + 10 μg of recombinant Pxn-1). Each group was immunized by subcutaneous injection in the dorsal region on days 0, 7 and 14 of pregnancy. Emulsified MPLA was produced according to [15].

### Course of pregnancy

The maternal weight gain, food and water intake were measured daily during the experiment. At day 21 of pregnancy, all rats were anesthetized using sodium pentobarbital and blood samples were collected by decapitation, transferred to anticoagulant-free test tubes, maintained on ice for 30 min and then centrifuged at 1300 ×G during 10 min at 4°C. The supernatant was collected as serum and stored at −80°C for further determination of immunological and biochemical parameters.

The uterus was removed and weighed and ovary and uterine contents were examined to determine the number of corpora lutea, implantation sites, resorptions (embryonic death), and the number of viable fetuses. The rate of embryonic loss before implantation was calculated as follows: (number of corpora lutea—number of implantations) x 100/number of corpora lutea. This calculation was used as a measurement of failed conception effects or preimplantation loss. The percentage of embryonic loss after implantation was calculated as follows: (number of implantations—number of live fetuses) x 100/number of implantations. This calculation was used as a measurement of the abortifacient effect or for identification of post implantation loss [23]. When a lack of visible implantation sites was observed, the uterine corns were stained with a preparation of 10% ammonium sulfate [24]. The fetuses and placentas were weighed to calculate the placental efficiency as fetal weight/placental weight [25].

### Analysis of external and internal (visceral and skeletal) anomalies

The fetuses were evaluated in a microscope with respect to incidence of external anomaly. After external analysis, half the fetuses were fixed in Bouin's fluid and serial sections were prepared as described by Wilson for visceral examination [26]. The remaining fetuses were prepared for examination of the bones by the staining procedure of Staples and Schnell [27]. Besides the skeletal analyses, the counting of the ossification sites was performed according to methodology proposed by Aliverti *et al*., which determines the degree of fetal development [28].

### Biochemical profile analysis

Serum concentrations of total cholesterol (CHO), triglycerides (TG) and high-density lipoprotein (HDL) were determined using the enzymatic method, and total protein (TP) concentrations, and aspartate aminotransferase (AST) and alanine aminotransferase (ALT) activities were estimated by the colorimetric method (Young, 2000). Very low-density lipoprotein (VLDL)-cholesterol levels were determined by mathematical estimation [29].

### Hematological analysis

For hematological analysis, blood was collected (500 μL) and transferred to tubes with anticoagulant (EDTA). The total leukocyte count was determined on blood samples diluted 1:20 in Turk’s solution using a Neubauer’s hemocytometer. For differential white blood cell counting, blood smears were fixed with methanol and stained with Giemsa’s solution. According to staining and morphological criteria, differential cell analysis was performed under the light microscope by counting 100 cells, and the percentage of each cell type was calculated.

### Antigen-specific antibody assays

Serum collected from all animals was assayed for the presence of peroxidoxin-specific imunoglobulin G (IgG) by enzyme-linked immunosorbent assay (ELISA). Briefly, 96-well microtiter plates were coated with peroxidoxin antigen diluted to 50ng/well in sodium carbonate buffer (pH 9.6) and incubated overnight at 4°C. The plates were blocked with 100 μL of 5% PBS-BSA for 1 h at 37°C and treated successively with 1:100 dilutions of the rats serum samples for 1 h at 37°C. Peroxidase-labeled antibodies specific to Rat IgG (Sigma-Aldrich, USA) were diluted at 1:3,000 and added for 1 h at 37°C. The wells were washed, and the chromogen solution—TMB substrate (Sigma-Aldrich, USA) diluted in citrate buffer containing hydrogen peroxide—was added and plates were incubated for 30 min in the dark. The enzymatic reaction was stopped by the addition of 4 N H_2_SO_4_, and the absorbance was determined at 450 nm on automatic microplate reader (Versamax, Molecular Devices, USA). Each sample was assayed in duplicate. The cutoff value was calculated using the mean average of the optical density of control group (0.171) plus 2 standard deviations (0.005 x2) to positive result. The cutoff was chosen based on the point that provides the maximum of the sum of the sensitivity and specificity [30].

### Statistical analysis

Comparison of the mean values between the experimental groups were determined by analysis of variance (ANOVA) followed by Tukey’s Multiple Comparison test. Differences in proportions were calculated by the Fisher´s Exact test. Differences were considered statistically significant when p<0.05.

## Results

**Fig 1:**
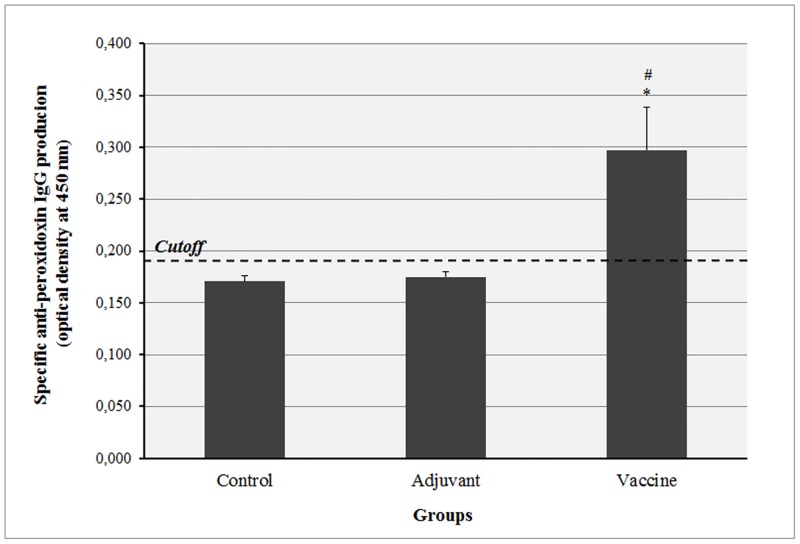
Quantification of maternal IgG anti-peroxidoxin of rats immunized with saline (Control), MPLA (Adjuvant) or MPLA + recombinant peroxidoxin of *Leishmania braziliensis* (Vaccine) during pregnancy. **Data is shown as mean ± Standard Deviation (SD)**. *p<0.*001 compared to Control group (ANOVA followed Tukey’s Multiple Comparison test)*. ^*#*^*p<0*.*001 compared to Adjuvant group (ANOVA followed by Tukey’s Multiple Comparison test)*

The quantification of maternal rat IgG anti-peroxidoxin after immunization was shown in Fig 1. Animals that received recombinant peroxidoxin formulated with emulsified MPLA presented was detectable in circulating IgG levels when compared to control groups (immunized with adjuvant or PBS alone), which did not presented production of specific anti-leishmanial antibodies.

A progressive increase in maternal weight gain during pregnancy were observed in all groups and exposure to the vaccine or adjuvant did not significantly alter the body weight of rats at any time. Food and water intake of animals (Table 1), biochemical (Table 2) and leukocyte profile (supplementary material) did not differ between groups during pregnancy.

**Table 1:** Body weight, water intake and food consumption of rats treated with saline (Control), MPLA (Adjuvant) or MPLA + recombinant peroxidoxin of *Leishmania braziliensis* (Vaccine) during pregnancy.

	*Groups*		
	*Control*	*Adjuvant*	*Vaccine*
***Body weight (g)***			
Day 0	257.2 ± 26.5	260.7 ± 20.4	263.1 ± 17.2
Day 7	274.7 ± 27.9	276.0 ± 23.9	279.0 ± 23.3
Day 14	298.2 ± 29.3	309.1 ± 25.7	312.1 ± 25.2
Day 20	363.5 ± 34.9	386.6 ± 41.1	376.1 ± 32.8
***Water intake (mL)***			
Day 0	30.0 ± 7.9	26.4 ± 9.2	28.2 ± 8.7
Day 7	35.4 ± 9.0	32.3 ± 11.7	30.9 ± 12.2
Day 14	35.8 ± 10.6	32.3 ± 11.3	35.9 ± 11.6
Day 20	36.8 ± 12.4	36.8 ± 9.5	43.6 ± 9.5
***Food consumption (g)***			
Day 0	14.1 ± 2.6	14.6 ± 3.5	14.6 ± 3.5
Day 7	19.1 ± 3.3	19.2 ± 2.1	17.4 ± 3.7
Day 14	19.9 ± 3.8	23.3 ± 3.6	21.4 ± 4.1
Day 20	19.8 ± 4.3	21.3 ± 4.7	21.2 ± 4.4

Data shown as mean± standard deviation (SD).

p>0.05 no significant difference (ANOVA followed by Tukey’s Multiple Comparison test)

**Table 2:** Biochemical parameters of rats treated with saline (Control), MPLA (Adjuvant) or MPLA + recombinant peroxidoxin of *Leishmania braziliensis* (Vaccine) during pregnancy.

	*Groups*		
	*Control*	*Adjuvant*	*Vaccine*
Total protein (g/dL)	3.4 ± 0.4	3.2 ± 0.4	3.4 ± 0.6
Triglycerides (mg/dL)	170.2 ± 97.2	177.3 ± 83.6	194.4 ± 134.7
Cholesterol (mg/dL)	84.1 ± 12.5	87.6 ± 14.1	86.5 ± 12.2
HDL (mg/dL)	43.0 ± 8.7	42.7 ± 7.4	51.0± 17.8
VLDL (mg/dL)	34.1 ± 19.4	35.5 ± 16.7	38.9 ± 26.9
ALT(U/L)	77.4 ± 22.5	67.1 ± 18.6	69.7 ± 11.8
AST (U/L)	207.6 ± 47.9	184.0 ± 47.9	189.6 ± 53.3

Data shown as mean± standard deviation (SD).

p>0.05 no significant difference (ANOVA followed by Tukey’s Multiple Comparison test)

The reproductive outcome of rats after immunization process is presented on Table 3. Animals from Vaccine group presented a significant increase in the rate of post implantation loss (14.7%) when compared to animals that received PBS or MPLA only (5.0 and 4.4%, respectively).

**Table 3:** Reproductive outcome from rats treated with saline (Control), MPLA (Adjuvant) or MPLA + recombinant peroxidoxin of *Leishmania braziliensis* (Vaccine) during pregnancy.

	Groups		
	*Control*	*Adjuvant*	*Vaccine*
Pregnant females (N)	13	11	11
Pregnant at term (N)	13	11	11
With total resorptions (N)	0	0	0
Corpora lutea			
• Total (N)	167	163	157
• Mean ± SD^a^	12.8 ± 2.1	14.8 ± 2.0	14.3 ± 1.8
Implantation			
• Total (N)	148	142	140
• Mean ± SD^a^	11.3 ± 3.1	12.9 ± 3.3	12.7 ± 2.2
Live fetuses			
• Total (N)	140	136	122
• Mean ± SD^a^	10.8±3.3	12.4 ± 3.2	11.1 ± 2.2
Dead fetuses			
• Total (N)	1	1	1
• Mean ± SD^a^	0.1 ± 0.3	0.1 ± 0.3	0.1 ± 0.3
Resorptions			
• Total (N)	7	5	17
• Mean ± SD^a^	0.5 ± 0.9	0.4 ± 0.5	1.5 ± 1.9
Pre-implantation loss (%)^b^	13.6	14.8	12.1
Post-implantation loss (%)^b^	5.0	4.4	14.7*^#^
Sex ratio (M/F)^b^	69/71	71/65	64/58
Maternal weight gain (g)^a^	106.3 ± 22.1	125.9 ± 23.4	113.0 ± 24.7
Gravid uterus weight (g)^a^	75.2 ± 16.2	89.6 ± 13.7	82.8 ± 17.3
Maternal weight gain minus Gravid uterus weight (g)^a^	34.6 ± 16.1	38.3 ± 20.7	30.2 ± 19.4
Fetal body weight (g)			
• Mean ± SD ^a^	5.1 ± 0.5	5.0 ± 0.6	5.2 ± 0.5
Placental weight (g)			
• Mean ± SD ^a^	0.5 ± 0.1	0.5 ± 0.1	0.5 ± 0.1
Placental efficiency			
• Mean ± SD^a^	10.5 ± 1.9	10.4 ± 1.6	10.5 ± 1.7

N = number. Data shown as mean± standard deviation (SD) and proportions (%).

*p<0.02 compared to Control group;

^#^p<0.01 compared to Adjuvant group (^a^ANOVA followed Tukey’s Multiple Comparison test;^b^Fisher Exact test)

Ossification sites (Table 4) and external anomalies frequency of fetuses (Table 5) did not present significant differences among the experimental groups. Changes in specific skeletal abnormalities were observed in animals that received the formulated vaccine, with a significant increase in incomplete ossification of sternebra in fetuses and abnormally shaped sternebra (when compared to Adjuvant group) and significant increased frequency of incomplete ossification of cranius (when compared to both Control and Adjuvant groups). However, there was no change in the total number of skeletal anomalies. Analysis of visceral anomalies showed that animals vaccinated with peroxidoxin presented the highest number of total alterations, including enlarged esophagus and trachea (not observed in the control groups) although no significant differences were detected among the experimental groups.

**Table 4:** Ossification sites of rats treated with saline (Control), MPLA (Adjuvant) or MPLA + recombinant peroxidoxin of *Leishmania braziliensis* (Vaccine) during pregnancy.

	Groups		
	*Control*	*Adjuvant*	*Vaccine*
Forepaw phalanx	3.46 ± 0.45	3.19 ± 1.01	3.13 ± 0.61
Metacarpus	3.87 ± 0.45	4.00 ± 0.00	4.00 ± 0.00
Hindpaw phalanx	1.16 ± 1.14	0.63 ± 0.71	0.84 ± 0.95
Metatarsus	4.93 ± 0.09	4.82 ± 0.30	4.91 ± 0.14
Caudal vertebra	3.73 ± 1.30	3.47 ± 0.61	3.87 ± 0.90
Sternebra	5.99 ± 0.04	5.98 ± 0.05	5.97 ± 0.06
Total	23.14 ± 2.63	22.09 ± 2.11	23.01 ± 2.28

Data shown as mean± standard deviation (SD).

p>0.05 no significant difference (ANOVA followed by Tukey’s Multiple Comparison test)

**Table 5:** Frequency of fetal anomalies of rats treated with saline (Control), MPLA (Adjuvant) or MPLA + recombinant peroxidoxin of *Leishmania braziliensis* (Vaccine) during pregnancy.

Variables	Groups			
	Control	Adjuvant	Vaccine	p-value^a^
**External anomalies**				
Number fetuses examined (litter)	140 (13)	136 (11)	122 (11)	
Total number of fetuses (%) with alteration	0 (0.0%)	3 (2.2%)	0 (0.0%)	>0.05
Mean % fetuses with alteration per litter (mean ± SD)	0.0 ± 0.0	2.3 ± 5.7	0.0 ± 0.0	
*Asymmetrical legs*	0 *(0*.*0%)*	1 *(0*.*7%)*	0 *(0*.*0%)*	>0.05
*gastroschisis*	0 *(0*.*0%)*	2 *(1*.*5%)*	0 *(0*.*0%)*	>0.05
**Skeletal anomalies**				
Number fetuses examined (litter)	76 (13)	72 (11)	66 (11)	
Total number of fetuses (%) with alteration	33 (43.42%)	33 (45.83%)	27(40.91%)	>0.05
Mean % fetuses with alteration per litter (mean ± SD)	46.4 ± 29.7	46.8 ± 21.1	39.1± 29.8	
*Incomplete ossification of cranius*	0 *(0*.*0%)*	0 *(0*.*0%)*	2 *(3*.*0%)*	>0.05
*Incomplete ossif*. *of vert*. *centrum*	1 *(1*.*3%)*	5 *(6*.*9%)*	1 *(1*.*5%)*	>0.05
*Abnormally shaped of vert*. *centrum*	1 *(1*.*3%)*	2 *(2*.*8%)*	4 *(6*.*1%)*	>0.05
*Bipartite ossif*. *of vert*. *centrum*	0 *(0*.*0%)*	1 *(1*.*4%)*	0 *(0*.*0%)*	>0.05
*Supranumerary rib*	10 *(13*.*1%)*	6 *(8*.*3%)*	11 *(16*.*7%)*	>0.05
*Wavy rib*	1 *(1*.*3%)*	0 *(0*.*0%)*	0 *(0*.*0%)*	>0.05
*Sternebra agenesis*	1 *(1*.*3%)*	1 *(1*.*4%)*	2*(3*.*0%)*	>0.05
*Incomplete ossif*. *of sternebra*	8 *(10*.*5%)*	19 *(26*.4%)*	8 *(12*.*1%)*	0.02
*Bipartite sternebra*	4 *(5*.*3%)*	2 *(2*.*8%)*	5 *(7*.*6%)*	>0.05
*Abnormally shaped sternebra*	31 *(43*.*0%)*	24 *(33*.*3%)*	14 *(21*.2%)*	0.004
**Visceral anomalies**				
Number fetuses examined (litter)	64 (13)	64 (11)	56 (11)	
Total number of fetuses (%) with alteration	21 (32.8%)	25 (39.1%)	32 (57.1%)*	0.01
Mean % fetuses with alteration per litter (mean ± SD)	33.7 ± 24.5	44.7 ± 29.4	55.2 ± 27.6	
*Hydroureter*	10 *(15*.*6%)*	16 *(25*.*0%)*	16 *(28*.*6%)*	>0.05
*Hydronephrosis*	4 *(6*.*2%)*	3 *(4*.*7%)*	11*(19*.6%)*^#^	*0.04^#^0.02
*Convoluted ureter*	6 *(9*.*4%)*	1 *(1*.*7%)*	4 *(7*.*1%)*	>0.05
*Distended bladder*	2 *(3*.*1%)*	5 *(7*.*8%)*	2 *(3*.*6%)*	>0.05
*Dilated trachea*	1 *(1*.*7%)*	0 *(0*.0%)*	1 *(1*.*8%)*	>0.05

*compared to Control group (^a^Fisher Exact test)

^#^compared to Adjuvant group (^a^Fisher Exact test)

## Discussion

Vaccines represent a promising measure for controlling infectious diseases, as leishmaniasis. Safety and induction of long-term immune memory are important characteristics in an anti-leishmanial vaccine [31]. The use of recombinant protein vaccines requires the association with adjuvants to obtain a protective effect and immune consequence T-helper cells and humoral responses [32]. The current study showed that vaccination using a leishmanial recombinant protein as a model did stimulate the production of specific IgG antibodies, demonstrating the potential antigenicity of the Pxn-1 when formulated with MPLA.

Pregnancy is characterized by a progressive increase in maternal weight gain, due to the growth of the fetus and its annexes (around 40%) and own adaptations of the body (the remaining 60%), and it is characterized by anabolism in early and catabolism in late pregnancy [33]. The use of certain substances can cause maternal toxicity and interfere with the progression of maternal weight. According to US Environmental Protection Agency, reduction in body weight or a decrease in mass gain may reflect a variety of responses, including systemic toxicity [34]. Changes in the consumption of water and food [35] and changes in organ weights are also clinical signs that indicate toxicity [36]. In the present study, no differences in these parameters among the experimental groups, suggesting that administration of vaccine or adjuvant alone did not cause maternal toxicity, which was also supported by similarity of the biochemical and leukocyte profile observed among the experimental groups.

Reproductive toxicity may occur due to any interference caused by some substance in males and females reproductive capacity either in the prenatal or postnatal period [37]. Depending on the gestational period in which the external agents come in contact with the maternal organism, this exposure can result in different responses ranging from an anti-implantation effect, functional or morphological changes, general retardation of development, malformations incidence to lethality [38]. All animals of this study showed pregnancy at term, and our results demonstrate that peroxidoxin vaccine, although induced important reproductive outcomes, it does not represent a lethal agent (at least in the dose used in the study) for pregnant females.

The corpora lutea and implantations were used to verify the effect of a particular environmental factor in the pre-implantation period (day 0 to 4 of pregnancy) or to evidence the anti-implantation effect of environmental factors [38]. These parameters did not differ among the groups, indicating that administration of the formulated vaccine did not cause anti-implantation effect. However, a significant increase in post-implantation loss rate in the vaccine group compared to other groups was observed, which might be associated with a possible pattern of immune response induced by vaccination. Of note, among the immunological factors that lead to pregnancy success is the modulation to T helper type 2 (Th2) immune response [39–41]. As the embryo in the maternal body behaves as a semi-allogenic graft, highly vulnerable to immune rejection and tolerance theories, the modulation for the Th2 response is crucial. Although not assessed in the present work, the modulation of the immune response by the vaccine towards a Th1 response might account as determinant factor that causes embryonic loss after the implantation process of the embryo.

The placental weight and placental efficiency were similar among groups. Placental efficiency is a measure to confirm the placental ability to ensure the maternal-fetal exchanges and the nutrient supply to the developing fetus [42]. The appropriate placental function was not affected by immunization and contributed to proper fetal development, as confirmed by similar weighting of fetuses. Furthermore, the analysis of the ossification centers of the fetuses from rats immunized with formulated vaccine or adjuvant alone, showed that the vaccination did not influence on fetal skeletal maturity. Chahoud and Paumgartten found a strong correlation between body weight and degree of ossification, which can be observed in our study [43].

There was no change in the frequency of total external and skeletal anomalies, and only a significant increase in incomplete ossification of sternebra of the adjuvant group fetuses and decreased asymmetric sternebra fetuses in the vaccine group. These skeletal abnormalities are considered as variations [44]. Abnormalities classified as variation do not affect the survival or health, and may disappear in the postnatal period. This may include a delay in growth or morphogenesis. Abnormalities classified as malformation causes permanent structural changes, which adversely affect the survival or health of the species under investigation [44,45]. Since there was no change in the total frequency of skeletal anomalies, these changes can be considered as transitory changes.

Regarding the visceral anomalies, the vaccine group showed an increase in the total frequency of these abnormalities. The hydronephrosis was the predominant abnormality in this group and this anomaly is considered a malformation [44,46]. Teratogenic processes in embryonic tissues include alteration in metabolic and systemic signaling [47], such as inositol metabolism [48], the polyol pathway [49], arachidonic acid /prostaglandin [50,51] and reactive oxygen species (ROS) [52,53]. The presence of Pxn-1 during pregnancy might influence the action of embryological signaling, especially in the renal system formation. Interestingly, previous studies demonstrated that Pxn-1, which is abundantly expressed in the amastigote phase of the *Leishmania* species [54], is important to detoxify reactive oxygen (ROS) and nitrogen (RNS) species promoting the parasite survival by enhancement of survival within macrophages [55]. Whether the reduction of host’s ROS or RNS activity by administration of Pxn-1 are still need to elucidated, the reactive oxygen species (ROS) and reactive nitrogen species (RNS) are important to regulate molecular and biochemical pathways responsible for the regulation of the survival of human monocytes, serving as important intracellular signaling molecules that influence cellular survival. Of note, human monocytes are influenced by intracellular production of ROS and RNS, which affects both monocyte survival and death [56].

Based on the results of this study, we conclude that the vaccine comprised of recombinant *Leishmania braziliensis*-derived Pxn-1 formulated with the *Bordetella pertussis* derived MPLA adjuvant is potentially antigenic when administered during pregnancy. Despite the absence of maternal toxicity, the use of the vaccine promoted increased embryonic losses after embryo implantation and increased frequency of visceral anomalies, showing that its use during pregnancy requires care and further study. Finally, we here demonstrated the adjuvant MPLA is safe and it might be used during maternal immunizations.

## Supporting information

S1 Table Peripheral blood cells of rats treated with saline (Control), MPLA (Adjuvant) or MPLA + recombinant peroxidoxin of Leishmania braziliensis (Vaccine) during pregnancy. (DOCX)
