# Supplementary material for: Safety evaluation of a vaccine: Effect in maternal reproductive outcome and fetal anomaly frequency in rats using a leishmanial vaccine as a model
Source: PLoS One. 2017 Mar 1;12(3):e0172525. doi: 10.1371/journal.pone.0172525 (PMC5332059; doi:10.1371/journal.pone.0172525)
Supplement: S1 Table — (DOCX) [file pone.0172525.s001.docx]

|  | ***Groups*** | | |
| --- | --- | --- | --- |
|  | *Control* | *Adjuvant* | *Vaccine* |
| White blood cells (10³/mm³) | 9.2 ± 1.4 | 8.5 ± 1.0 | 9.6 ± 1.0 |
| Neutrophil (%) | 36.4 ± 7.8 | 35.7 ± 3.4 | 35.8 ± 4.4 |
| Eosinophil (%) | 0.4 ± 0.9 | 0.8 ± 1.1 | 0.5 ± 1.4 |
| Lymphocyte (%) | 60.1 ± 6.5 | 61.1 ± 4.4 | 61.8 ± 4.4 |
| Monocyte (%) | 2.6 ± 0.5 | 1.7 ± 0.8 | 1.8 ±1.4 |
